# Combined Dielectric-Optical Characterization of Single Cells Using Dielectrophoresis-Imaging Flow Cytometry

**DOI:** 10.3390/bios14120577

**Published:** 2024-11-27

**Authors:** Behnam Arzhang, Justyna Lee, Emerich Kovacs, Michael Butler, Elham Salimi, Douglas J. Thomson, Greg E. Bridges

**Affiliations:** 1 Department of Electrical and Computer Engineering, University of Manitoba, Winnipeg, MB R3T 5V6, Canada; arzhang1@myumanitoba.ca (B.A.); leej16@myumanitoba.ca (J.L.); kovacse3@myumanitoba.ca (E.K.); elham.salimi@umanitoba.ca (E.S.); douglas.thomson@umanitoba.ca (D.J.T.); 2 National Institute for Bioprocessing Research and Training, A94 X099 Dublin, Ireland; michael.butler@nibrt.ie

**Keywords:** Chinese hamster ovary (CHO), dielectrophoresis, dielectric spectroscopy, microfluidics, microscopy, single cell analysis, flow cytometry

## Abstract

In this paper, we present a microfluidic flow cytometer for simultaneous imaging and dielectric characterization of individual biological cells within a flow. Utilizing a combination of dielectrophoresis (DEP) and high-speed imaging, this system offers a dual-modality approach to analyze both cell morphology and dielectric properties, enhancing the ability to analyze, characterize, and discriminate cells in a heterogeneous population. A high-speed camera is used to capture images of and track multiple cells in real-time as they flow through a microfluidic channel. A wide channel is used, enabling analysis of many cells in parallel. A coplanar electrode array perpendicular to cell flow is incorporated at the bottom of the channel to perform dielectrophoresis-based dielectric characterization. A frequency-dependent voltage applied to the array produces a non-uniform electric field, translating cells to higher or lower velocity depending on their dielectric polarizability. In this paper, we demonstrate how cell size, obtained by optical imaging, and DEP response, obtained by particle tracking, can be used to discriminate viable and non-viable Chinese hamster ovary cells in a heterogeneous cell culture. Multiphysics electrostatic-fluid dynamics simulation is used to develop a relationship between cell incoming velocity, differential velocity, size, and the cell’s polarizability, which can subsequently be used to evaluate its physiological state. Measurement of a mixture of polystyrene microspheres is used to evaluate the accuracy of the cytometer.

## 1. Introduction

For clinical and diagnostic applications, rapid discrimination and characterization of single biological cells within a large heterogeneous population is desired [1,2]. Microfluidic devices are ideal platforms for this, as they provide rapid single cell analysis [3,4,5,6,7,8] and have been used for cell identification, analysis, separation, and manipulation [9,10,11,12,13,14,15,16]. Optical and dielectric analysis are two different modalities and are usually performed independently using different hardware. Optical imaging analysis provides information on cell morphological parameters such as size, surface roughness, eccentricity, and nucleus size [17,18,19,20,21,22]. Dielectric analysis, performed at frequencies in the beta-dispersion region, provides information on plasma membrane complexity and permeability, net ion concentration in the cytoplasm and nucleus, and even the presence of smaller membrane bound organelles [22,23,24,25,26,27]. RF and microwave impedance sensing have been combined with optical imaging to characterize pollen grains and yeast cells [28,29]. Combined, optical and dielectric analysis can provide a large array of independent parameters, enabling researchers to determine an individual cell’s physiological state or discriminate cell phenotype with improved precision.

Optical imaging and dielectric spectroscopy are two of many techniques used for cell analysis. Dielectrophoresis (DEP) is a label-free and non-invasive method based on the cell’s dielectric properties [7,8,9,10,11,12,15,16,22,23,25,26,30,31,32,33]. For example, dielectrophoretic field-flow fractionation is an established technique for separating and discriminating different cell types [34,35]. Dielectrophoresis operates by applying a spatially non-uniform electric field to a polarizable particle such as a biological cell. This results in asymmetric forces being exerted on the induced dipole, generating a dielectrophoretic force causing translation of the cell as it passes through the field. Cell motion induced by dielectrophoresis (DEP) is influenced by the frequency and spatial configuration of the applied electric field, the dielectric properties of the surrounding medium, and the dielectric properties of the cell itself [25,36], as described through the Clausius-Mossotti factor (CMF). The CMF is a frequency-dependent parameter dependent on the dielectric properties of the cell’s membrane and internal compartments and of the surrounding medium [37,38]. Thus, how a cell responds to the DEP force can provide information about its structure, function, and health [20,26,39,40,41,42].

In-flow optical image analysis uses a microscopy system with a high-speed camera to capture real-time images of cells flowing in the microfluidic channel as they pass through a detection window. The technique analyzes each cell’s image and attributes properties such as size, eccentricity, and surface roughness [19,43]. One of the advantages of in-flow optical analysis is the capacity to rapidly record the optical properties of a large number of cells, enhancing the understanding of cellular heterogeneity. For example, the approach has been used in bioprocessing monitoring to classify cells as viable, necrotic, or apoptotic based on detailed morphological analysis [19]. Viable cells typically have an intact and uniform shape, necrotic cells exhibit signs of membrane damage and irregular morphology, and apoptotic cells can be identified by specific features such as cell shrinkage and surface blebbing.

In this paper, we present a dual-modality DEP-imaging cell analysis microfluidic system that provides information on both the morphology and dielectric properties of every cell in a heterogeneous sample population. Particle imaging has previously been used in microfluidic systems that employ dielectrophoresis separation and analysis [44,45,46,47,48]. For more accurate particle dielectric analysis, we introduce a differential velocity DEP approach using particle tracking information [49]. Details of the DEP-imaging cytometer are described, along with a multiphysics electrostatic-fluid dynamics model that enables accurate extraction of the Clausius-Mossotti factor of individual cells. The analysis model incorporates size information from optical analysis to enhance dielectric characterization. We evaluate the device using a mixture of different-sized polystyrene microspheres to extract fluid dynamics parameters. We apply the device to analyze a Chinese hamster ovary cell culture grown in nutrient-depleted conditions, demonstrating the ability to discriminate a population of both viable and non-viable cells.

## 2. Materials and Methods

### 2.1. Device Description and Operation

**Figure 1:**
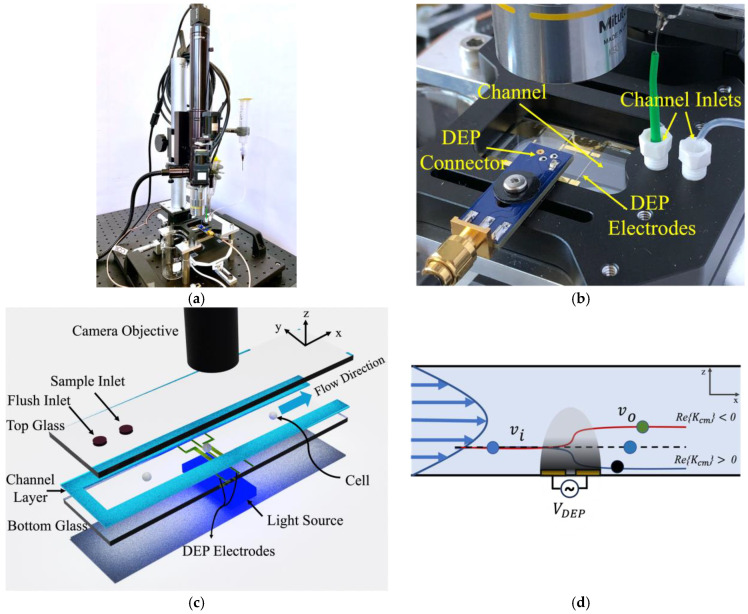
(**a**) Experimental setup of the DEP-imaging flow cytometer. (**b**) Microfluidic chip, fluid delivery, and interface for DEP. (**c**) Schematic diagram of the DEP-imaging flow cytometer, comprising camera, light source, and microfluidic channel sandwiched between glass slides with fluid inlet ports. (**d**) Longitudinal cross-section indicating cell trajectory in a parabolic laminar flow and showing height and velocity change induced by DEP actuation due to the non-uniform field above coplanar electrodes.

The in-flow DEP-imaging flow cytometer is illustrated in Figure 1. The device includes a microfluidic channel, light source, and high-speed camera. A 50 µm deep, 8 mm wide channel is fabricated using double-sided tape sandwiched between two 1 mm × 25 mm × 75 mm glass slides. Two gold coplanar electrodes, 35 µm wide with a 25 µm gap, are patterned on the bottom slide for application of DEP. The electrodes are perpendicular to cell flow and run the entire width of the channel. The DEP actuation voltage, $V_{DEP}$, is applied at contacts at the edge of the chip. Two fluid ports are drilled into the top slide. Cells enter at one port and are imaged as they flow over the DEP actuation region. A flushing inlet enables cleaning of the channel. As in [49,50], a wide channel is used to provide a large imaging area enabling analysis of many cells in parallel.

Analysis samples with a concentration ranging from $5\times 10^{4}$ to $5\times 10^{5}$ particles/mL are pumped through the channel using a gravity feed approach. An average fluid velocity of 1300 µm/s, corresponding to a volumetric flow rate of 30 µL/min, is typically used. A FLIR Blackfly™ (BFS-U3-16S2C-CS, Edmund Optics, Barrington, NJ, USA) camera with a frame rate of 226 fps and a 1.6 MP sensor is used to capture images over a 670 × 894 µm^2^ field of view, corresponding to a 0.62 µm/pixel resolution. This allows for processing with a maximum throughput of over 20 particles/s under ideal conditions. An image processing and cell tracking algorithm, based on Track.py [51], uses static background subtraction to identify and track moving cells and a defined size threshold to ignore small objects, such as debris. Information, including position, velocity, diameter, and eccentricity for each cell, is analyzed. Differential velocity ${(v}_{i}-v_{o})$, before and after the DEP electrodes is used for dielectric analysis. Change in velocity is due to change in cell height within the channel as a result of DEP actuation as shown in Figure 1d.

#### 2.1.1. Cell Tracking Algorithm

**Figure 2:**
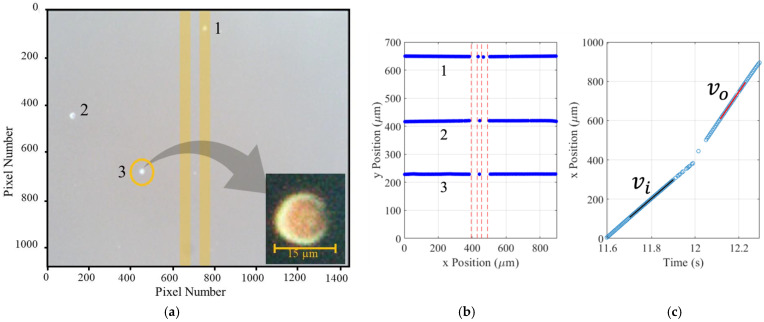
Optical capture and analysis of cells. Background subtraction, contrast enhancement, bright spot elimination, and cell visibility enhancement are used to track cells and identify key cell features. (**a**) Example of one gray-scaled video frame. The CHO cell in the inset is 23.7 pixels across (the electrodes are manually added in this plot). (**b**) The trajectory of cells is plotted based on data obtained by the tracking algorithm. (**c**) Tracking data are used to plot position vs time and find the cell’s velocity before and after the electrode.

Our system illuminates cells from below in quasi-darkfield mode. As the optical refractive index of biological cells is very similar to that of the suspension medium, this helps to illuminate the cells and enhance the cell’s edge features. A Gaussian filter is applied to each video frame to increase the contrast between the cells and their background. The filtered frame serves as a reference to distinguish the static background, which is subsequently subtracted, retaining only cells. A threshold is then applied, amplifying the brightness of the cells within the frame. As the frame is processed, key features are identified, including the centroid of each cell, cell eccentricity, area, and diameter. Features that deviate significantly in terms of size or have an unusually high eccentricity (identifying debris or aggregated cells) are labeled as anomalies and excluded. An example single frame is shown in Figure 2a, containing three cells. The inset is 39×39 pixels showing a cell with a 14.7 µm diameter and eccentricity of 0.57.

Once all frames have been processed, cell motion is analyzed using Track.py. The trajectory and position of the three cells in Figure 2a are shown in Figure 2b as they are tracked across the camera’s field of view and through the DEP actuation region. For a typical input velocity of $v_{i}=1000\;\mu m/s$, individual data points correspond to capturing cell displacements of approximately 4.4 μm/frame (see Supplementary Materials for details). The input and output velocities ${(v}_{i}$ and $v_{o})$ are derived by calculating the slope of the best-fit line to the cell’s centroid prior to and after encountering the DEP electrodes, as shown in Figure 2c. Change in velocity is used to determine the dielectric characteristics of the cell, as discussed in the following sections.

#### 2.1.2. Dielectrophoresis Analysis

In-flow dielectrophoresis is used to determine the dielectric characteristics of cells. A polarizable particle, such as a cell, will experience a DEP force when exposed to a non-uniform electric field. The particle’s polarizability and the surrounding medium’s permittivity determine the DEP force’s amplitude and direction. The time-average DEP force for a spherical particle of radius, r, is determined by [25,52]

(1) $${\vec{F}}_{DEP}=2\pi \varepsilon_{0}\varepsilon_{rm}r^{3}Re\left\{K_{cm}\left(\omega \right)\right\}\nabla {\left|E_{rms}^{DEP}\right|}^{2}.$$

Here, $E_{RMS}^{DEP}$ is the rms value of the electric field at the particle’s location due to the sinusoidal voltage, $V_{DEP}$, applied to the electrodes as shown in Figure 1. $Re\left\{K_{cm}(\omega )\right\}$ is the real part of the Clausius-Mossotti factor, given as

(2) $$K_{cm}=\frac{{\tilde{\varepsilon }}_{p}-{\tilde{\varepsilon }}_{m}}{{\tilde{\varepsilon }}_{p}+{2\tilde{\varepsilon }}_{m}},$$

where ${\tilde{\varepsilon }}_{p}$ and ${\tilde{\varepsilon }}_{m}$ are the complex permittivity of the particle and medium, ${\tilde{\varepsilon }}_{p}=\varepsilon_{0}\varepsilon_{rp}-j\sigma_{p}/\omega$ and ${\tilde{\varepsilon }}_{m}=\varepsilon_{0}\varepsilon_{rm}-j\sigma_{m}/\omega$, where ω is angular frequency, $\sigma_{p}$ and $\sigma_{m}$ are the particle and medium conductivity, and $\varepsilon_{rp}$ and $\varepsilon_{rm}$ are the relative permittivity of the particle and medium, respectively.

**Figure 3:**
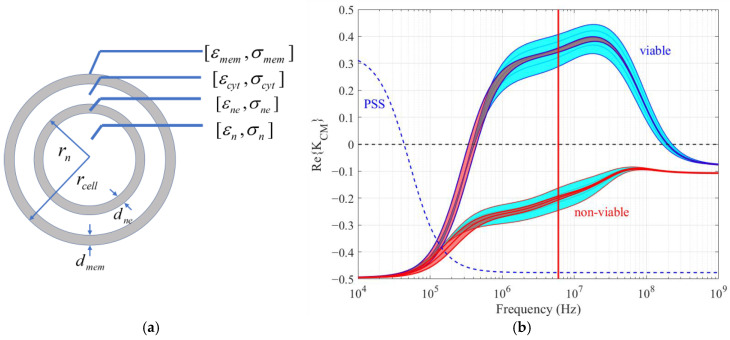
(**a**) Double-shell model of a CHO cell with radius $r_{cell}$. (**b**) $Re\left\{K_{cm}(\omega )\right\}$ spectra for viable and non-viable CHO cells in a medium with $\sigma_{med}=0.17\;S/m$ and $\varepsilon_{med}=78\;\varepsilon_{0}$. Nominal size and electrical parameters are taken from [42] and provided in Table 1. The red regions show the effect of ±20% variation in the cell size ($r_{cell}=5–7.5\;m$ for viable cells and $r_{cell}=4.5–7\;m$ for non-viable cells). The cyan regions show the effect of ±20% variation in the cytoplasm conductivity ($\sigma_{cyt}=0.43-0.63\;S/m$ for viable cells and $\sigma_{cyt}=0.055-0.085\;S/m$ for non-viable cells). The dashed line shows $Re\left\{K_{cm}(\omega )\right\}$ for a 15.7 µm diameter PSS with $\varepsilon_{rb}\;=\;2.5$ and surface conductance, $K_{surf}\;=\;1$ nS in a DI water medium [57].

The real part of $K_{cm}$ is frequency dependent and exhibits a dispersive behavior dependent on the dielectric properties of the particle [9,24,42]. A biological cell’s complex permittivity depends on both its structure and the dielectric characteristics of its internal organelles, cytoplasm, and plasma membrane. A two-shell model, as shown in Figure 3a, is an effective model that represents most of the dispersion processes in the beta-dispersion frequency range (100 kHz–500 MHz) [42]. It includes the plasma membrane, cytoplasm, and nucleus. Smaller organelles can be incorporated using an appropriate effective medium theory. The double-shell model is commonly used to model the $Re\{K_{cm}\}$ spectrum for a wide range of cell types and consists of eight dielectric and four geometric factors [53]. Figure 3b shows the $Re\left\{K_{cm}\right\}$ spectrum for CHO cells as well as a polystyrene microsphere. Nominal values for the geometric and electrical parameters are taken from ref. [42], which are based on extensive measurements, and are listed in Table 1. If the DEP frequency is chosen appropriately, the sign and magnitude of $Re\{K_{cm}\}$ can be used to identify cell phenotype, such as viable and non-viable cells, as indicated by the 6 MHz red line in Figure 3b [41,42]. The effect of a ±20% change in cell size (red band) and cytoplasm conductivity (cyan band) are also shown in Figure 3b. This shows that viable-non-viable discrimination is possible even with large cell-to-cell heterogeneity.

The dielectric differences between viable and non-viable cells as indicated in Table 1 are primarily a reflection of differences in cell membrane permittivity and conductivity, cytoplasm and nucleus conductivity, and cell and nucleus size [42]. As cells progress from viable to non-viable, their size decreases [39,54]. During starvation, cell membrane active ion pump function reduces, leading to changes in cytoplasm ion concentration and a subsequent decrease in cytoplasm conductivity [39,55,56], this occurring during the early stages of apoptosis. During later-stage apoptosis, the cell membrane smooths, blebs form, and cell membrane integrity is compromised [19,43]. This results in a decrease in membrane permittivity and an increase in membrane conductivity [39,42]. The effects of size and conductivity change on the Clausius-Mossotti factor are shown in Figure 3b. Membrane permittivity change closely follows size change. We see that cytoplasm conductivity change provides an excellent indicator of viability.

#### 2.1.3. Dielectric-Fluid Dynamics Model

Device operation is illustrated in Figure 1d. The distribution of cells entering the DEP actuation region is close to their equilibrium height $h_{i}$ and velocity $v_{i}$. Cells exit at a different height, $h_{o}$, and velocity, $v_{o}$, due to DEP forces at the electrodes and hydrodynamic forces [33,52]. The real component of the Clausius-Mossotti factor, *Re*{$K_{cm}$} (Equation (1)), determines the direction and amplitude of the DEP force. If the DEP force is attractive, *Re*{$K_{cm}$} is positive (pDEP), and a cell will translate downward, resulting in a decrease in height and velocity. Alternatively, when *Re*{$K_{cm}$} is negative (nDEP), a cell will be repelled from the electrodes, resulting in an increase in height and velocity. Differential velocity, $v_{i}-v_{o}$, input velocity $v_{i}$, and cell size are obtained by optical particle tracking and can be mapped to *Re*{$K_{cm}$} using multiphysics simulation. Further, as described in [38,42], the two-shell compartment dielectric properties for a certain cell phenotypic may be determined by measuring the $K_{cm}(\omega )$ spectrum over a range of frequency. This may also be utilized for determining physiological aspects such as cytoplasm conductivity, which is related to ion concentration and mobility in the cytoplasm, and membrane capacitance, which is related to membrane complexity.

In this paper, multiphysics simulation using COMSOL™ fluid dynamics is used to model the cell trajectory. The net force on a particle includes buoyancy, gravity, lift, drag, and dielectrophoresis (DEP) forces [33,52,58] as follows:

(3) $${\vec{F}}_{total}={\vec{F}}_{DEP}+{\vec{F}}_{drag}+{\vec{F}}_{grav}+{\vec{F}}_{buoy}+{\vec{F}}_{lift}.$$

Here, ${\vec{F}}_{DEP}$ was given by Equation (1), and gravity and buoyancy forces are

(4) $${\vec{F}}_{grav}+{\vec{F}}_{buoy}=\frac{4}{3}\pi gr^{3}(\rho_{p}-\rho_{m})\hat{z},$$

where g is the gravitational acceleration constant with $\rho_{p}$ and $\rho_{m}$, the mass densities of particle and medium, respectively. The hydrodynamic lift force is approximated as

(5) $${\vec{F}}_{lift}=\frac{6C\eta r^{3}\langle v\rangle sgn(z)}{H(H/2-\left|z\right|-r)}\hat{z},$$

where C is a lift force constant, H is the height of the channel, z=H/2−h;(−H/2+r≤z≤H/2−r), and h is the distance from the bottom of the channel to the center of the particle. Here, $\langle v\rangle$ is the average velocity of the fluid in the channel, and η is the viscosity of the medium. The drag force is

(6) $${\vec{F}}_{drag}=\left\{\begin{array}{c} 6\pi r\eta ({\vec{v}}_{mx}-{\vec{v}}_{px})\hat{x}\\ 6\pi r\eta ({\vec{v}}_{mz}-{\vec{v}}_{pz})\lambda \hat{z} \end{array}\right.,$$

where ${\vec{v}}_{m}$ and ${\vec{v}}_{p}$ are the velocity of medium and particle, respectively. Here, λ is the ratio of the force that a particle experiences when it translates perpendicular to two confining parallel walls compared to the force in an unbounded fluid. Its value is a function of the cell’s altitude in the channel and varies as the cell responds to the DEP force. For the geometry of the channel, the altitude-dependent λ for our system is determined using the theoretical model [59]. For our device, the cell size-to-height ratio may be small, and the λ factor can be significant. Flow in the channel is assumed laminar with a parabolic velocity profile [58], so that at a height, h, the fluid velocity ${\vec{v}}_{m}$ is

(7) $${\vec{v}}_{m}=6\langle v\rangle \frac{h}{H}\left(1-\frac{h}{H}\right)\hat{x}.$$

The channel geometry and fluid dynamics parameters used for our device are provided in Table 2.

### 2.2. Cell Preparation

Cell growth and preparation processes are detailed in [39,41]; however, a brief summary is provided here. Chinese hamster ovary cells (CHODG44-EG2-hFc/clone 1A7), provided by Yves Durocher of the National Research Council, are grown in 250 mL shaker flasks and incubated at 37 °C with a 10% CO_2_ overlay on a shaker platform (120 rpm). Cells are passaged with a seeding density of 2.5 × 10^5^ cells/mL in BioGro-CHO™ serum-free medium (BioGro Technologies, Winnipeg, MB, Canada) and supplemented with 0.5 g/L yeast extract (BD, Sparks, MD, USA), 1 mM glutamine (Sigma-Aldrich, St. Louis, MO, USA), and 4 mM GlutaMax I (Invitrogen, Grand Island, NY, USA). To produce a population containing both viable and non-viable cells, CHO cells are seeded and kept for five days, during which time their viability is monitored. For DEP measurements of samples containing both viable and non-viable cells, an appropriate volume of day five cells was removed from the culture and added to a mix of BioGro CHO medium and low conductivity medium [22.9 mM sucrose (Sigma-Aldrich, St. Louis, MO, USA), 16 mM glucose (Thermo Fisher Scientific, Waltham, MA, USA), 1 mM CaCl_2_ (Thermo Fisher Scientific, Waltham, MA, USA), 16 mM Na_2_HPO_4_ (Thermo Fisher Scientific, Waltham, MA, USA)] with a 1:15 ratio. A 20 mL sample was obtained with a concentration of 5 × 10^4^ cells/mL and conductivity of 0.17 S/m. Measurements were conducted within 20 min of resuspending the cells in the low conductivity DEP medium to minimize the impact on cells. A trypan blue assay was used to determine cell viability, and a cell counter (Countess™ 3 FL, Thermo Fisher Scientific, Waltham, MA, USA) was used to measure the size and size distributions of both viable and non-viable cells.

## 3. Results

### 3.1. Evaluation Using PSS

In our experiments, the optical imaging region is centered over the electrode array so that particles can be tracked and analyzed before and after DEP actuation. As explained in the previous section, this can be mapped to the sign and amplitude of $Re\left\{K_{cm}\right\}$. Polystyrene microspheres (PSS) (Polysciences™, Warrington, PA, USA [61]) suspended in deionized (DI) water have permittivity, ${\tilde{\varepsilon }}_{b}=\varepsilon_{0}\varepsilon_{rb}-j2_{surf}/r\omega$, with $\varepsilon_{rb}\;\sim \;2.5$ and surface conductance, $K_{surf}\;\sim \;1$ nS [57]. PSS are homogeneous dielectric spheres and due to the surface conductance have a simpler dispersive behavior as compared with biological cells, as shown in Figure 3b. For an applied DEP frequency of 1 MHz, PSS in DI water have a real part of the Clausius-Mossotti factor $Re\left\{K_{cm}\right\}\approx -0.5$ for sizes similar to many biological cells (5–20 μm diameter) and are used to evaluate our system.

**Figure 4:**
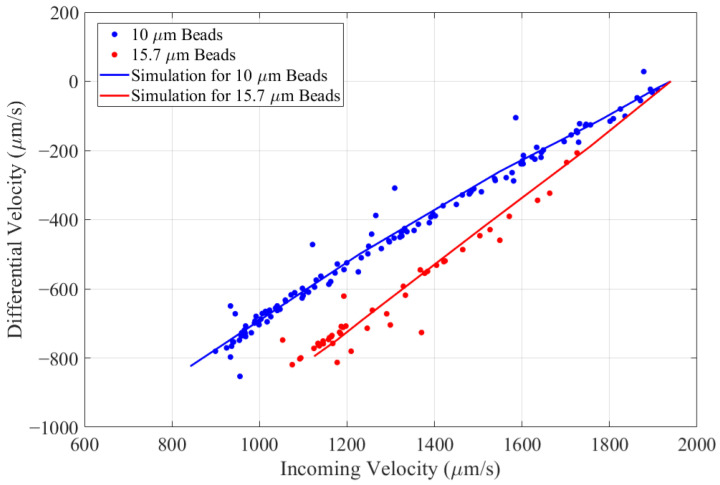
Differential velocity ${(v}_{i}-v_{o})$ versus incoming velocity for a mixture of 10 µm (blue) and 15.7 µm (red) polystyrene microspheres at V_DEP_ = 6 V_pp_ and f = 1 MHz. Data points are colored according to optically measured size (see Figure 5).

Figure 4 shows the differential velocity versus incoming velocity for a two-population mixture of 10 µm and 15.7 µm polystyrene microspheres for a DEP voltage 6 V_pp_ at f=1 MHz. Clear differentiation between the two populations can be made. The differential velocities calculated by multiphysics simulation using the parameters in Table 1 are given by solid curves. This shows that a mapping from (incoming velocity, differential velocity, and size) as obtained by optical particle tracking to a unique value of $Re\left\{K_{cm}\right\}$ can be made for each particle.

**Figure 5:**
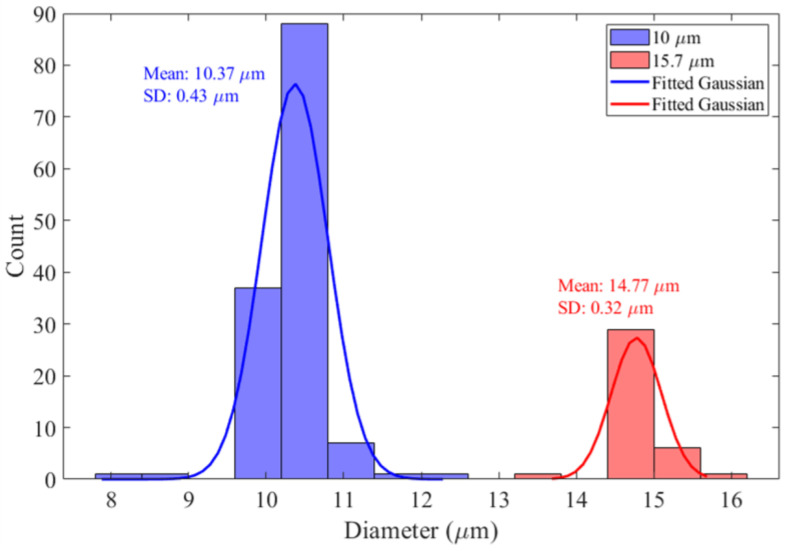
Particle size distribution for imaged 10 µm (blue) and 15.7 µm (red) diameter PSS corresponding to the colored data points in Figure 4.

Figure 5 gives the particle size distribution from optical image analysis corresponding to the data points in Figure 4 for the mixture of 10 µm and 15.7 µm PSS. The process of determining particle size involves edge detection following a threshold process, effectively separating the particles from the background and creating an image of each particle. In correspondence with the DEP differential velocity data shown in Figure 4, two distinct populations are observed. The fitted normal distributions have means of 10.37 µm and 14.77 µm with standard deviations of 0.43 µm and 0.32 µm, respectively. The corresponding manufacturer’s (Polysciences™ [61]) size standard deviations are 0.8 µm and 1.41 µm, respectively.

### 3.2. Analysis of Viable and Non-Viable CHO Cells

**Figure 6:**
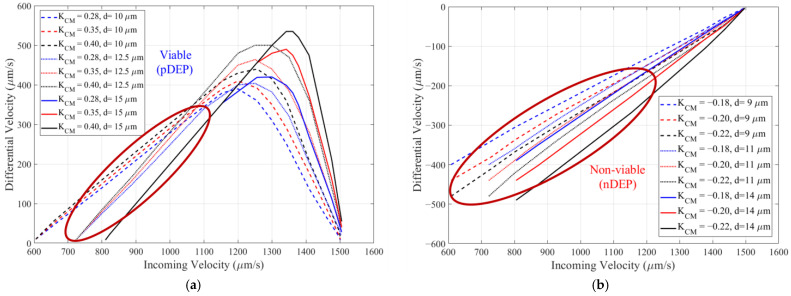
Simulation of the differential velocity, ${(v}_{i}-v_{o})$, as a function of incoming velocity, $v_{i}$, for cells with diameters and Clausius-Mossotti factor values typical of (**a**) viable and (**b**) non-viable cells. A 20% variation in $Re\left\{K_{cm}\right\}$ and size is evaluated.

Measuring cell viability provides important information on the physiological state of a cell when monitoring its response to environmental stress, drug treatment, or external stimuli [26,41,56,62]. It is of particular importance in bioprocessing, where monitoring viable cell density in a culture is used for feeding strategies and determining optimal harvest times [19,63]. To determine the optimal operating conditions of our device for discriminating viable and non-viable Chinese hamster ovary cells, an initial multiphysics simulation is performed. The dielectric parameters for viable/non-viable CHO from Table 1 along with the fluid dynamics parameters from Table 2 are used. A DEP voltage $V_{DEP}=8\;V_{pp}$ and a frequency of 6 MHz are used as viable cells exhibit pDEP and non-viable cell exhibit nDEP. Figure 6a,b show the simulated differential velocity as a function of incoming velocity for viable cells and non-viable cells, respectively. A 20% variation of $Re\left\{K_{cm}\right\}$ at 6 MHz, based on Figure 3b, is considered. For viable cells, the range [0.28–0.4] for $Re\left\{K_{cm}\right\}$ is used, and for non-viable cells, the range [−0.22–−0.18] is used. A 20% variation in size, [10–15 µm] for viable cells and [9–14 µm] for non-viable cells, is also considered. This range of size is typical of what is measured using a Countess™ 3 FL cell counter.

Figure 6 provides an indication of how the differential velocity is expected to vary for different cell sizes and $Re\left\{K_{cm}\right\}$, which is important when the device is used to discriminate between two different cell phenotypes (see Appendix A). Additionally, it is a mapping that can be used to determine the value of $Re\left\{K_{cm}\right\}$ based on the measured cell size, incoming velocity, and differential velocity that our device provides. This can be used to determine quantitative dielectric values for the cells, but this is not the focus of this paper. The incoming velocity varies depending on the cell size and how the cells enter the channel through the inlet port of the device (see Figure 1c). Therefore, a distribution of incoming and differential velocities is expected in the experimental results. The simulations show that cell size variation should not dramatically affect the discrimination capability of the DEP-imaging flow cytometer approach. For maximizing the difference in differential velocity between viable and non-viable cells, Figure 6 suggests that the device should be operated such that the incoming velocity is in the range of 800–1200 μm/s.

**Figure 7:**
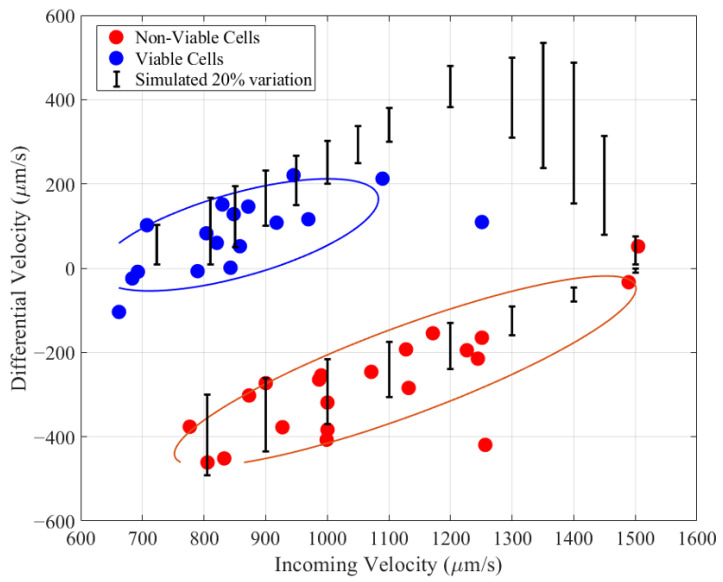
Scatter plot of differential velocity versus incoming velocity for CHO cells for a DEP frequency of 6 MHz. A Gaussian mixture model clustering algorithm is used to separate CHO cell populations into viable (blue) and non-viable (red) clusters. The bars indicate the simulated variation due to 20% variability in cell size and $Re\left\{K_{cm}\right\}$.

Cell preparation and experiment procedure follow a previously developed protocol where Chinese hamster ovary cells were cultivated and kept for 5 days in a BioGro-CHO™ medium in order to produce a sample with both viable and non-viable cells. After 120 h, cells were extracted and resuspended in a medium with conductivity σ=0.17 S/m and immediately measured using the DEP-imaging cytometer for a DEP voltage of 8 V_pp_ (see Supplementary Materials). Cell viability was 70% as measured by trypan blue assay. The differential velocity versus incoming velocity for each cell for a DEP frequency of 6 MHz is shown in Figure 7 (see Appendix A). The experiment was conducted for an input velocity corresponding to the circled areas in Figure 6.

The two distinct populations observed in Figure 7 are identified as viable and non-viable cells according to the CMF spectrum shown in Figure 3b. A Gaussian mixture model clustering algorithm, based on differential and incoming velocities, is employed to identify the two populations within the experimental data. The high degree of separation between clusters demonstrates the device’s ability to discriminate cells based on their dielectric properties. The ellipsoids surrounding each cluster in Figure 7 represent a defined probability that a data point belongs to that cluster’s Gaussian distribution (71% for viable cells—blue and 75% for non-viable cells—red).

Figure 7 shows a larger number of non-viable cells than viable cells (45% viability), which is lower than that measured by trypan blue assay (70% viability). This discrepancy matches other dielectric-based measurements of viability, which showed that the dielectric response of cells follows early apoptotic events, whereas trypan blue is a measure of late apoptosis [41]. The vertical bars in Figure 7 show the range of differential velocity as obtained by simulation for a 20% variation in $Re\left\{K_{cm}\right\}$ value and a 20% variation in cell size as shown in Figure 6a for viable cells (pDEP) and Figure 6b for non-viable cells (nDEP). The measured DEP response for frequency 3 MHz as well as for no applied DEP voltage is provided in the Appendix A. The no-DEP measurement indicates that the device has a differential velocity measurement standard deviation of 56 μm/s.

**Figure 8:**
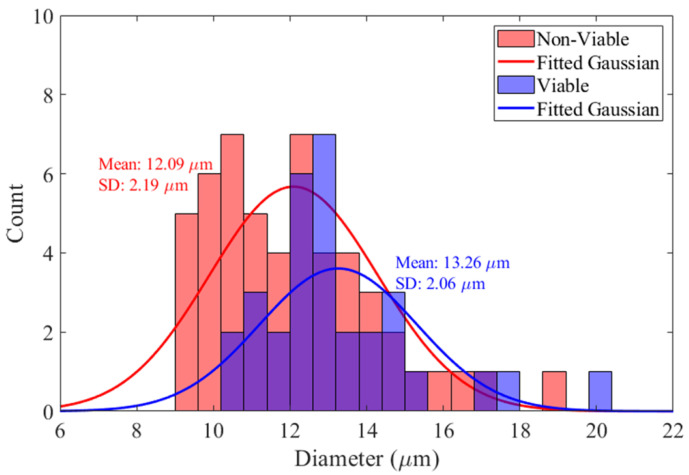
Size distributions for viable and non-viable CHO cells, as obtained by combining the cluster data in Figure 7 at 6 MHz and in Figure A1 at 3 MHz.

**Figure A1:**
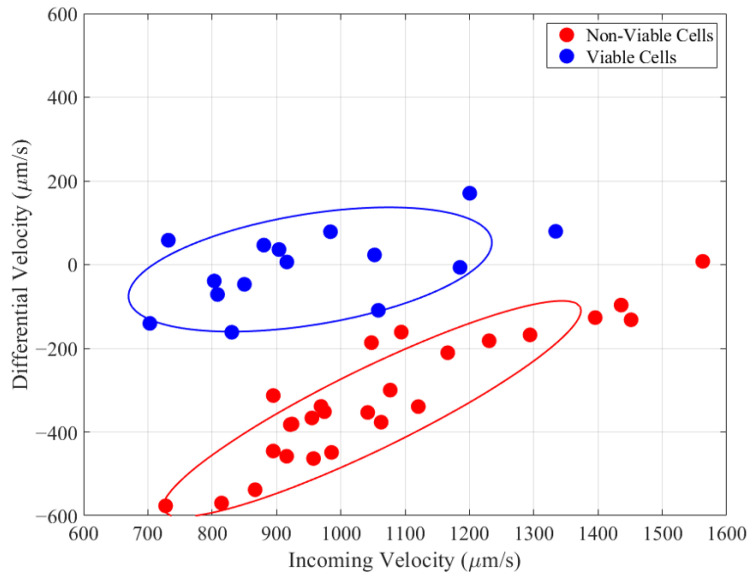
Scatter plot of differential velocity versus incoming velocity for CHO cells for a DEP frequency of 3 MHz. A Gaussian mixture model clustering algorithm is used to segregate CHO cell populations into viable (blue) and non-viable (red) clusters.

Figure 8 shows the distributions of cell sizes obtained by optical analysis of viable and non-viable CHO cell clusters. Size distribution data are from the viable and non-viable clusters in both Figure 7 (measured at 6 MHz) and Figure A1 (measured at 3 MHz). The analysis shows a discernible difference in size between the two cell populations, with viable cells exhibiting a marginally larger mean diameter compared to non-viable cells. This is in line with previous experiments [39,42]. The decrease is due to the physiological changes that a cell undergoes during apoptosis reflecting cellular structure or integrity alterations [54,64] and aligns with the observation that viable cells generally maintain a larger volume [65]. In conjunction with DEP, the size information can serve as an additional parameter for enhancing the accuracy of label-free cell viability.

**Figure 9:**
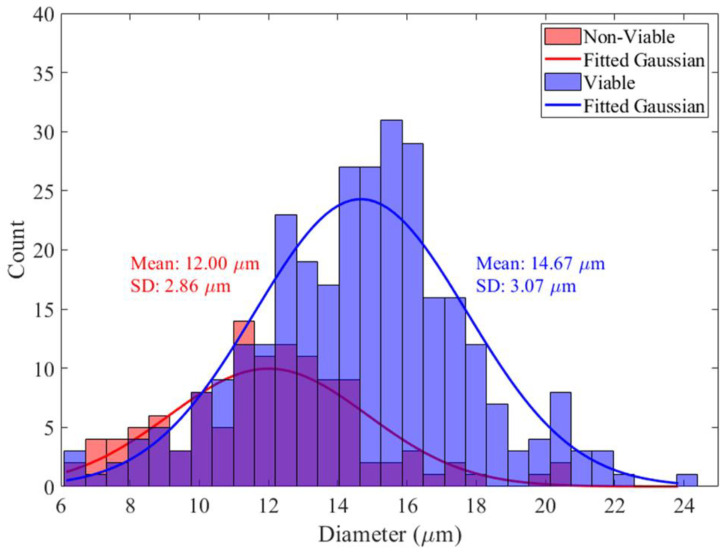
Size distributions for CHO-EG2 cells measured by a cell counter. A viability of 70% was measured using trypan blue assay.

For comparison, the size distributions of cells and their viability using a trypan blue assay for the same cell sample as used for DEP measurements (measured using a cell counter Countess™ 3 FL) are shown in Figure 9. The fitted normal distributions have means of 12.00 µm and 14.67 µm, with standard deviations of 2.86 µm and 3.07 µm for non-viable and viable cells, respectively. The fitted normal distributions shown in Figure 8, obtained from the DEP-imaging flow cytometer, have means of 12.09 µm and 13.26 µm, with standard deviations of 2.19 µm and 2.06 µm for non-viable and viable cells, respectively. The smaller standard deviations obtained by the DEP-imaging flow cytometer suggest more accurate and consistent-in-size measurements. It should be noted that the DEP-imaging cytometer measures cell size when in suspended flow, while the cell counter measures cell size after they have settled on the surface of a counting slide.

## 4. Conclusions

In this paper, we introduced a microfluidic cytometer that enables simultaneous dielectric characterization and microscopic imaging of single biological cells while in flow. By integrating dielectric characterization with high-throughput imaging within a microfluidic framework, the device provides a novel method for multimodal label-free, non-invasive analysis of cellular properties. If the DEP frequency and flow velocity are chosen judiciously, discrimination of cell phenotype is possible. We demonstrated that for CHO cells, a high degree of confidence in identifying viable and non-viable (apoptotic) cells is possible. Analysis of cell size using complimentary imaging data provides additional information when discriminating cell phenotype. In the case of CHO cell culture, monitoring a decrease in average size would indicate that the cell population viability was in decline, supporting DEP measurements.

A multiphysics model was developed, enabling the mapping of incoming velocity, differential velocity, and cell size—derived from image analysis and particle tracking—to the Clausius-Mossotti factor of each individual cell. Subsequently, the dielectric properties could be mapped to physiological properties such as cell membrane complexity or internal cell ion concentrations as done in [39]. The additional information on cell size is important in enhancing the mapping accuracy.

The current device represents a robust proof of concept, and there is potential for further improvements. The throughput can be increased dramatically by optimizing sample cell density and the incoming flow velocity. A higher-resolution camera with a higher frame rate would enable more precise cell tracking and provide improved cell imaging. More importantly, since the optical system has a limited depth of focus, it is essential to keep cells within this range of height to accurately extract their features. Adding DEP electrodes on the top surface of the microfluidic channel would focus incoming cells within a controlled height range. This would ensure that incoming cells flow through the channel with more uniform velocity, enhancing the consistency of both dielectric and imaging analyses. Additionally, DEP-imaging of fluorescent labeled cells could be used to improve specificity and provide a method of correlating specific cell functions with dielectric properties [66].

Our dielectric-optical cytometer can play an important role in bioprocessing monitoring and control. Knowledge of when cell cultures enter an early apoptotic phase can influence feeding strategies or aid in deciding when to terminate a process before cell lysis, reducing the risk of early host cell protein release. We demonstrated that DEP measurement of CHO cells at 6 MHz can distinguish viable and non-viable cells and, at this frequency, is most significantly related to differences in cytoplasm conductivity. Change in cytoplasm conductivity occurs during early apoptosis before cell membrane integrity is compromised. Additionally, in conjunction with individual cell size information from optical measurement, viable cell count can be used to accurately quantify viable-cell density in a culture.

## Figures and Tables

**Table 1:** Dielectric parameters for viable and non-viable CHO cells [42].

**Parameter**	**Symbol**	Viable CHO	Non-Viable CHO
Average particle radius (μm)	$r_{cell}$	6.25	5.5
Nucleus radius (μm)	$r_{n}$	$0.55*r_{cell}$	$0.55*r_{cell}$
Nuclear envelope thickness (nm)	$d_{n}$	40	40
Plasma membrane thickness (nm)	$d_{mem}$	5	5
Membrane conductivity (S/m)	$\sigma_{mem}$	$1\times 10^{-6}$	$1\times 10^{-6}$
Nuclear envelope conductivity (S/m)	$\sigma_{ne}$	$1\times 10^{-3}$	$1\times 10^{-3}$
Nuclear envelope permittivity (F/m)	$\varepsilon_{ne}$	$11.5\varepsilon_{0}$	$11.5\varepsilon_{0}$
Cytoplasm permittivity (F/m)	$\varepsilon_{cyt}$	$54.5\varepsilon_{0}$	$54.5\varepsilon_{0}$
Cytoplasm conductivity (S/m)	$\sigma_{cyt}$	0.53	0.07
Membrane permittivity (F/m)	$\varepsilon_{mem}$	$8.5\varepsilon_{0}$	${5\;\varepsilon }_{0}$
Nucleus conductivity (S/m)	$\sigma_{n}$	1.5	0.56
Nucleus permittivity (F/m)	$\varepsilon_{n}$	$120\varepsilon_{0}$	$69\varepsilon_{0}$

**Table 2:** Fluid dynamics parameters for PSS and CHO cells.

Parameter	Symbol	PSS	CHO
Average particle radius (μm)	r	5–7.5	4.5–7.5
Channel height (μm)	H	50	50
Medium density (Kg/m^3^)	$\rho_{m}$	1000	1015–1020
Medium viscosity (Pas) [60]	η	0.001	0.001
Average flow velocity (μm/s)	$\langle v\rangle$	1300	1000–1200
Particle density (Kg/m^3^)	$\rho_{p}$	1050	1050
Lift coefficient [38]	C	0.31	0.31

**Figure A2:**
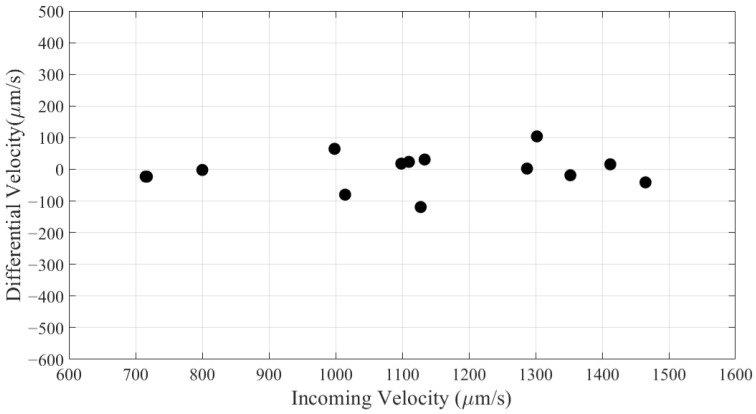
Differential velocity versus incoming velocity for CHO cells when $V_{DEP}=0\;V$ (no DEP).

## Data Availability

Data are contained within the article.

## Supplementary Materials

The following supporting information can be downloaded at https://www.mdpi.com/article/10.3390/bios14120577/s1, Figure S1: Imaging video frames, with cells highlighted by yellow circles. (a) Frame showing three cells in the imaging region and (b) a later frame showing cell positions as they traverse to the right. (c) Processed frame corresponding to (a), where detected cells are shown in yellow. (d) Processed frame corresponding to (b); Figure S2: Trajectories of detected Cells. This figure illustrates the positions of cells within the field of view, with each cell tracked throughout the video to analyze motion in response to experimental conditions; Video S1: Video of the mixture of viable/non-viable CHO cells passing the field of view, utilized for data extraction and analysis of trajectories and differential velocities; Video S2: Video of the mixture of PSS passing the field of view, utilized for data extraction and analysis of trajectories and differential velocities.

## Appendix A

The differential velocity versus incoming velocity for each cell for a DEP frequency of 3 MHz is shown in the scatter plot of Figure A1. Similar to Figure 7, experimental conditions are the same as described in Section 3.2 for a CHO cell culture with a viability of 70% as measured by trypan blue assay. The experiment was conducted for a flow velocity corresponding to the circled areas in Figure 6. A Gaussian mixture model clustering algorithm is used to identify viable and non-viable cells. Combined cell size data corresponding to the clusters in Figure A1 and Figure 7 are used to produce the size distributions in Figure 8.

Figure A2 shows the differential velocity versus incoming velocity when $V_{DEP}=0\;V$ (no-DEP case) is applied to the device electrodes. The result shows a mean differential velocity of −3.37 µm/s with a standard deviation of 55.9 µm/s.

## References

[1] Han A., Yang L., Frazier A.B. (2007). Quantification of the Heterogeneity in Breast Cancer Cell Lines Using Whole-Cell Impedance Spectroscopy. Clin. Cancer Res..

[2] Givan A.L. (2001). Flow Cytometry: First Principles.

[3] Zhou W.-M., Yan Y.-Y., Guo Q.-R., Ji H., Xu T.-T., Makabel B., Pilarsky C., He G., Zhang J.-Y. (2021). Microfluidics Applications for High-Throughput Single Cell Sequencing. J. Nanobiotechnol..

[4] Yi C., Li C.-W., Ji S., Yang M. (2006). Microfluidics Technology for Manipulation and Analysis of Biological Cells. Anal. Chim. Acta.

[5] Wang D., Bodovitz S. (2010). Single Cell Analysis: The New Frontier in ‘Omics’. Trends Biotechnol..

[6] Taheri-Araghi S., Brown S.D., Sauls J.T., McIntosh D.B., Jun S. (2015). Single-Cell Physiology. Annu. Rev. Biophys..

[7] Lambert E., Manczak R., Barthout E., Saada S., Porcù E., Maule F., Bessette B., Viola G., Persano L., Dalmay C. (2021). Microfluidic Lab-on-a-Chip Based on UHF-Dielectrophoresis for Stemness Phenotype Characterization and Discrimination among Glioblastoma Cells. Biosensors.

[8] Giduthuri A.T., Theodossiou S.K., Schiele N.R., Srivastava S.K. (2021). Dielectrophoretic Characterization of Tenogenically Differentiating Mesenchymal Stem Cells. Biosensors.

[9] Pethig R. (2017). Dielectrophoresis: Theory, Methodology and Biological Applications.

[10] Wang X.-B., Huang Y., Gascoyne P.R.C., Becker F.F. (1997). Dielectrophoretic Manipulation of Particles. IEEE Trans. Ind. Appl..

[11] Valero A., Braschler T., Renaud P. (2010). A Unified Approach to Dielectric Single Cell Analysis: Impedance and Dielectrophoretic Force Spectroscopy. Lab Chip.

[12] Farasat M., Aalaei E., Ronizi S.K., Bakhshi A., Mirhosseini S., Zhang J., Nguyen N.-T., Kashaninejad N. (2022). Signal-Based Methods in Dielectrophoresis for Cell and Particle Separation. Biosensors.

[13] Sun T., Morgan H. (2010). Single-Cell Microfluidic Impedance Cytometry: A Review. Microfluid. Nanofluidics.

[14] Park H., Kim D., Yun K.S. (2010). Single-Cell Manipulation on Microfluidic Chip by Dielectrophoretic Actuation and Impedance Detection. Sens. Actuators B Chem..

[15] Gagnon Z.R. (2011). Cellular Dielectrophoresis: Applications to the Characterization, Manipulation, Separation, and Patterning of Cells. Electrophoresis.

[16] Yao J., Zhao K., Lou J., Zhang K. (2024). Recent Advances in Dielectrophoretic Manipulation and Separation of Microparticles and Biological Cells. Biosensors.

[17] Heo Y.J., Lee D., Kang J., Lee K., Chung W.K. (2017). Real-Time Image Processing for Microscopy-Based Label-Free Imaging Flow Cytometry in a Microfluidic Chip. Sci. Rep..

[18] Vedhanayagam A., Basu A.S. Imaging Flow Cytometry at >13K Events/S Using GPU-Accelerated Computer Vision. Proceedings of the 2019 IEEE SENSORS.

[19] Breen L., Flynn J., Bergin A., Flampouri E., Butler M. (2024). Single Cell Analysis of Chinese Hamster Ovary Cells During a Bioprocess Using a Novel Dynamic Imaging System. Biotechnol. Prog..

[20] Secme A., Tefek U., Sari B., Pisheh H.S., Uslu H.D., Çalıskan Ö.A., Kucukoglu B., Erdogan R.T., Alhmoud H., Sahin O. (2023). High-Resolution Dielectric Characterization of Single Cells and Microparticles Using Integrated Microfluidic Microwave Sensors. IEEE Sens. J..

[21] Mir M., Wang Z., Shen Z., Bednarz M., Bashir R., Golding I., Prasanth S.G., Popescu G. (2011). Optical Measurement of Cycle-Dependent Cell Growth. Proc. Natl. Acad. Sci. USA.

[22] Elitas M., Islam M., Korvink J.G., Sengul E., Sharbati P., Ozogul B., Kaymaz S.V. (2022). Quantifying Deformation and Migration Properties of U87 Glioma Cells Using Dielectrophoretic Forces. Biosensors.

[23] Su H.W., Prieto J.L., Voldman J. (2013). Rapid Dielectrophoretic Characterization of Single Cells Using the Dielectrophoretic Spring. Lab Chip.

[24] Asami K. (2002). Characterization of Heterogeneous Systems by Dielectric Spectroscopy. Prog. Polym. Sci..

[25] Jones T.B. (1995). Electromechanics of Particles.

[26] Wang X., Becker F.F., Gascoyne P.R.C. (2002). Membrane Dielectric Changes Indicate Induced Apoptosis in HL-60 Cells More Sensitively than Surface Phosphatidylserine Expression or DNA Fragmentation. Biochim. Biophys. Acta Biomembr..

[27] Polevaya Y., Ermolina I., Schlesinger M., Ginzburg B.Z., Feldman Y. (1999). Time Domain Dielectric Spectroscopy Study of Human Cells. Biochim. Biophys. Acta Biomembr..

[28] DaOrazio M., Reale R., De Ninno A., Brighetti M.A., Mencattini A., Businaro L., Martinelli E., Bisegna P., Travaglini A., Caselli F. (2022). Electro-Optical Classification of Pollen Grains via Microfluidics and Machine Learning. IEEE Trans. Biomed. Eng..

[29] Dahal N., Ehrett C., Osterberg J.A., Divan R., Wang P. (2023). Candida Cell Heterogeneity Measured with a Microwave Flow Cytometer. IEEE J. Electromagn. RF Microw. Med. Biol..

[30] Yan S., Yuan D. (2021). Continuous Microfluidic 3D Focusing Enabling Microflow Cytometry for Single-Cell Analysis. Talanta.

[31] Wang L., Lu J., Marchenko S.A., Monuki E.S., Flanagan L.A., Lee A.P. (2009). Dual Frequency Dielectrophoresis with Interdigitated Sidewall Electrodes for Microfluidic Flow-Through Separation of Beads and Cells. Electrophoresis.

[32] Giesler J., Weirauch L., Thöming J., Baune M., Pesch G.R. (2021). Separating Microparticles by Material and Size Using Dielectrophoretic Chromatography with Frequency Modulation. Sci. Rep..

[33] Afshar S., Salimi E., Braasch K., Butler M., Thomson D.J., Bridges G.E. (2016). Multi-Frequency DEP Cytometer Employing a Microwave Sensor for Dielectric Analysis of Single Cells. IEEE Trans. Microw. Theory Tech..

[34] Markx G.H., Rousselet J., Pethig R. (1997). DEP-FFF: Field-flow fractionation using non-uniform electric fields. J. Liq. Chromatogr. Relat. Technol..

[35] Wang X.B., Yang J., Huang Y., Vykoukal J., Becker F.F. (2000). Gascoyne PRC. Cell separation by dielectrophoretic field-flow-fractionation. Anal. Chem..

[36] Wang Y., Du F., Baune M., Thöming M. (2014). Dielectrophoresis in Aqueous Suspension: Impact of Electrode Configuration. Microfluid. Nanofluidics.

[37] Pethig R., Markx G.H. (1997). Applications of Dielectrophoresis in Biotechnology. Trends Biotechnol..

[38] Salimi E., Braasch K., Butler M., Thomson D.J., Bridges G.E. (2016). Dielectric Model for Chinese Hamster Ovary Cells Obtained by Dielectrophoresis Cytometry. Biomicrofluidics.

[39] Afshar S., Salimi E., Fazelkhah A., Braasch K., Mishra N., Butler M., Thomson D.J., Bridges G.E. (2019). Progression of Change in Membrane Capacitance and Cytoplasm Conductivity of Cells during Controlled Starvation Using Dual-Frequency DEP Cytometry. Anal. Chim. Acta.

[40] Pethig R., Kell D.B. (1987). The Passive Electrical Properties of Biological Systems: Their Significance in Physiology, Biophysics, and Biotechnology. Phys. Med. Biol..

[41] Salimi E., Braasch K., Fazelkhah A., Afshar S., Saboktakin Rizi B., Mohammad K., Butler M., Bridges G.E., Thomson D.J. (2018). Single Cell Dielectrophoresis Study of Apoptosis Progression Induced by Controlled Starvation. Bioelectrochemistry.

[42] Afshar S., Fazelkhah A., Braasch K., Salimi E., Butler M., Thomson D.J., Bridges G.E. (2021). Full Beta-Dispersion Region Dielectric Spectra and Dielectric Models of Viable and Non-Viable CHO Cells. IEEE J. Electromagn. RF Microw. Med. Biol..

[43] Gascoyne P.R.C., Shim S., Noshari J., Becker F.F., Stemke-Hale K. (2013). Correlations between the Dielectric Properties and Exterior Morphology of Cells Revealed by Dielectrophoretic Field-Flow Fractionation. Electrophoresis.

[44] Jeon H., Lee D.-H., Jundi B., Pinilla-Vera M., Baron R.M., Levy B.D., Voldman J., Han J. (2021). Fully Automated, Sample-to-Answer Leukocyte Functional Assessment Platform for Continuous Sepsis Monitoring via Microliters of Blood. ACS Sens..

[45] Fikar P., Georgiev V., Lissorgues G., Holubova M., Lysak D., Georgiev D. (2018). 2DEP Cytometry: Distributed Dielectrophoretic Cytometry for Live Cell Dielectric Signature Measurement on Population Level. Biomed. Microdevices.

[46] Godino N., Pfisterer F., Gerling T., Guernth-Marschner C., Duschl C., Kirschbaum M. (2019). Combining Dielectrophoresis and Computer Vision for Precise and Fully Automated Single-Cell Handling and Analysis. Lab Chip.

[47] Henslee E.A. (2020). Review: Dielectrophoresis in Cell Characterization. Electrophoresis.

[48] Chen S., Zhang S., Zhu R. (2022). Computer-Vision-Based Dielectrophoresis Mobility Tracking for Characterization of Single-Cell Biophysical Properties. Anal. Chem..

[49] Arzhang B., Lee J., Dietrich J., Absalan S., Kovacs E., Salimi E., Thomson D., Bridges G. Dielectrophoresis Characterization of Particles and Cells Using Imaging Flow Cytometry. Proceedings of the 2023 IEEE MTT-S International Conference on Numerical Electromagnetic and Multiphysics Modeling and Optimization (NEMO).

[50] Fazelkhah A., Afshar S., Durham N., Butler M., Salimi E., Bridges G., Thomson D. (2020). Parallel Single-Cell Optical Transit Dielectrophoresis Cytometer. Electrophoresis.

[51] Trackpy (Version 0.6.0). https://github.com/soft-matter/trackpy.

[52] Gascoyne P.R.C., Vykoukal J. (2002). Particle Separation by Dielectrophoresis. Electrophoresis.

[53] Cottet J., Fabregue O., Berger C., Buret F., Renaud P., Frénéa-Robin M. (2019). MyDEP: A New Computational Tool for Dielectric Modeling of Particles and Cells. Biophys. J..

[54] Model M.A., Schonbrun E. (2013). Optical Determination of Intracellular Water in Apoptotic Cells. J. Physiol..

[55] Panayiotidis M.I., Bortner C.D., Cidlowski J.A. (2006). On the Mechanism of Ionic Regulation of Apoptosis: Would the Na^+^/K^+^-ATPase Please Stand Up?. Acta Physiol..

[56] Mulhall H.J., Cardnell A., Hoettges K.F., Labeed F.H., Hughes M.P. (2015). Apoptosis Progression Studied Using Parallel Dielectrophoresis Electrophysiological Analysis and Flow Cytometry. Integr. Biol..

[57] Honegger T., Berton K., Picard E., Peyrade D. (2011). Determination of Clausius-Mossotti Factors and Surface Capacitances for Colloidal Particles. Appl. Phys. Lett..

[58] Shames I.H. (2003). Mechanics of Fluids.

[59] Ganatos P., Weinbaum S., Pfeffer R.A. (1980). A Strong Interaction Theory for the Creeping Motion of a Sphere Between Plane Parallel Boundaries. Part 1. Perpendicular Motion. J. Fluid Mech..

[60] Hofmann G. (1977). Iscotables: A Handbook of Data for Biological and Physical Scientists.

[61] Polybead^®^ Polystyrene Microspheres. https://www.polysciences.com/media/pdf/technical-data-sheets/238-Polystyrene-FAQ.pdf.

[62] Kasarabada V., Ahamed N.N.N., Vaghef-Koodehi A., Martinez-Martinez G., Lapizco-Encinas B.H. (2024). Separating the Living from the Dead: An Electrophoretic Approach. Anal. Chem..

[63] Opel C.F., Li J., Amanullah A. (2010). Quantitative Modeling of Viable Cell Density, Cell Size, Intracellular Conductivity, and Membrane Capacitance in Batch and Fed-Batch CHO Processes Using Dielectric Spectroscopy. Biotechnol. Prog..

[64] Kerr J.F.R., Wyllie A.H., Currie A.R. (1972). Apoptosis: A Basic Biological Phenomenon with Wide-ranging Implications in Tissue Kinetics. Br. J. Cancer.

[65] Kasim N.R., Kuželová K., Holoubek A., Model M.A. (2013). Live Fluorescence and Transmission-through-Dye Microscopic Study of Actinomycin D-Induced Apoptosis and Apoptotic Volume Decrease. Apoptosis.

[66] Winer M.H., Ahmadi A., Cheung K.C. (2014). Application of a Three-Dimensional (3D) Particle Tracking Method to Microfluidic Particle Focusing. Lab Chip.

