# Supplementary material for: Combined Dielectric-Optical Characterization of Single Cells Using Dielectrophoresis-Imaging Flow Cytometry
Source: Biosensors (Basel). 2024 Nov 27;14(12):577. doi: 10.3390/bios14120577 (PMC11674913; doi:10.3390/bios14120577)
Supplement: Supplementary file 1 [file biosensors-14-00577-s001.zip › biosensors-3300933-supplementary.pdf]

biosensors

MDPI

Supplementary Material

Combined Dielectric-Optical Characterization of Single Cells using Dielectrophoresis-Imaging Flow Cytometry

Behnam Arzhang [1], Justyna Lee [1], Emerich Kovacs [1], Michael Butler [2], Elham Salimi [1], Douglas J. Thomson [1] and Greg E. Bridges [1*]

[1] Department of Electrical and Computer Engineering, University of Manitoba, Winnipeg, MB, Canada, R3T 5V6; arzhang1@myumanitoba.ca (B.A.); leej16@myumanitoba.ca (J.L.); kovacse3@myumanitoba.ca (E.K.); elham.salimi@umanitoba.ca (E.S.); douglas.thomson@umanitoba.ca (D.J.T.)

[2] National Institute for Bioprocessing Research and Training, Dublin, Ireland, A94 X099; michael.butler@nibrt.ie

* Correspondence: gregory.bridges@umanitoba.ca

Experiment details for CHO cell analysis (Figure 7):

Figure 7 shows a scatter plot analysis of differential velocity versus incoming velocity of CHO cells for a 6 MHz DEP frequency. The analysis identifies two distinct populations, correlating with viable and non-viable cells, as classified in Figure 3(b). Cell viability was determined to be 70% using a trypan blue assay. This visualization helps differentiate cell viability based on the cells' response to DEP forces. The plot presents data captured from two 30-second videos totaling 60 seconds of observation, as analyzed using our DEP-imaging flow cytometer. For analysis, cells are removed from a day five culture and suspended in a low conductivity DEP measurement medium. During measurements, cell density was approximately 5×104 cells/mL. From Fig. 7, the average flow rate in the microfluidic channel is 1000 μm/s. The channel cross-section dimensions are 50 μm in height and 8000 μm in width, resulting in a volumetric flow rate of 0.4×10-3 mL/s. This corresponds to approximately 20 cells/s traversing through the entire channel cross section. However, the imaging system field of view is 670x894 μm, resulting in an approximate 2 cells/s throughput for imaging analysis. The 30-second imaging videos are substantial in size. Video S1 (CHO_Figure7_Video.mp4) in the supplementary material is a 10-second segment extracted from the original imaging videos. This segment contains 13 cells, of which six are effectively captured by our tracking algorithm and analyzed. Fig. S1 shows two examples of captured and analyzed cells. Fig. S1(a,b) show two example frames from the original video. Fig. S1(c,d) show the corresponding frames with processing applied (background subtraction and cell identification).

Citation: To be added by editorial staff during production.

Received: date
Revised: date
Accepted: date
Published: date

Copyright: © 2024 by the authors. Submitted for possible open access publication under the terms and conditions of the Creative Commons Attribution (CC BY) license (https://creativecommons.org/licenses/by/4.0/).

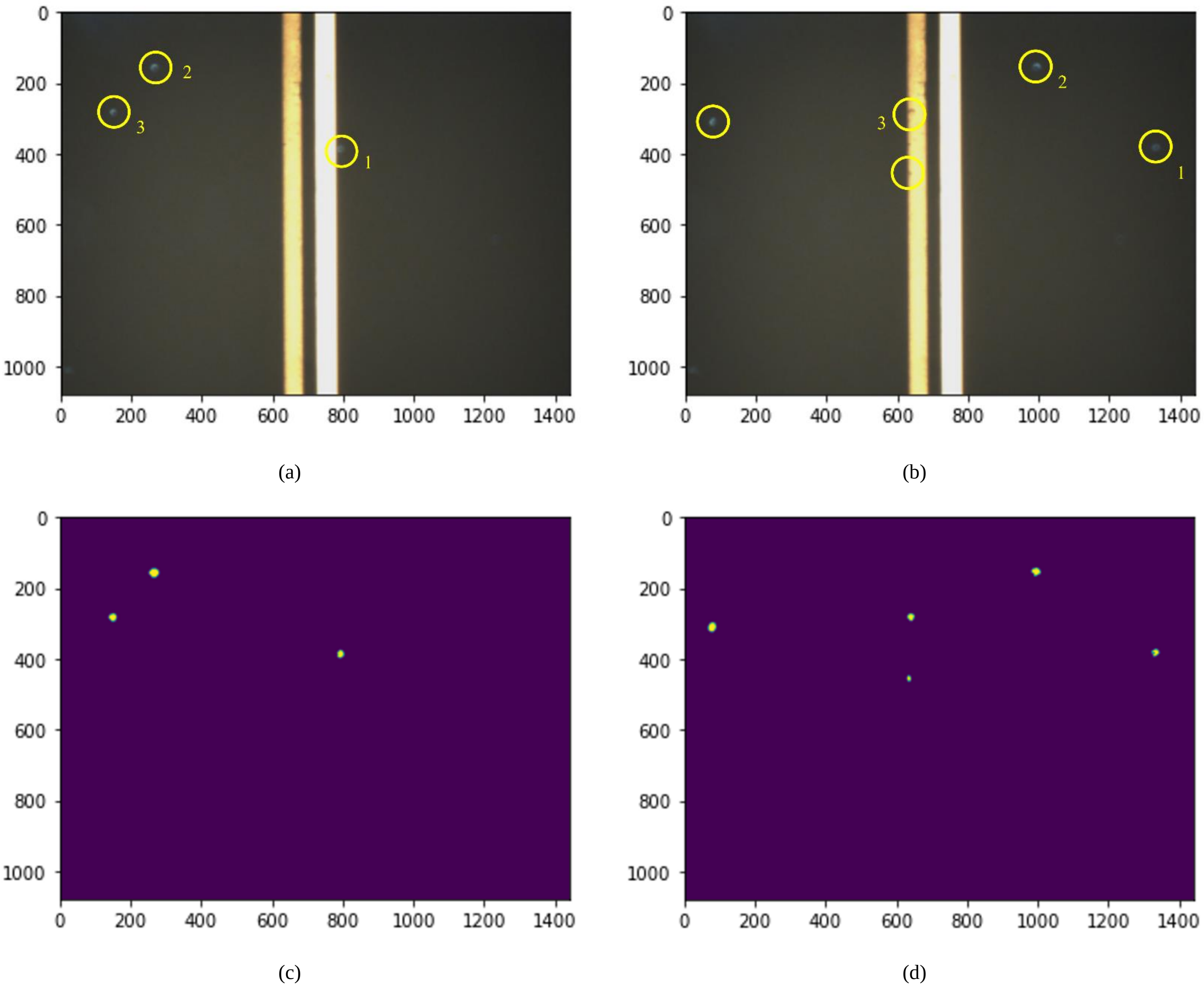

Figure S1. Imaging video frames, with cells highlighted by yellow circles. (a) Frame showing three cells in the imaging region and (b) a later frame showing cell positions as they traverse to the right. (c) Processed frame corresponding to (a), where detected cells are shown in yellow. (d) Processed frame corresponding to (b).

Figure S2 shows the (x,y) positions of tracked cells within the imaging system field of view corresponding to the supplementary Video S1 (CHO_Figure7_Video.mp4). The tracked (x,y) positions for each cell are analyzed before and after the DEP electrodes to provide their input and output velocities (v_i and v_o) and differential velocity as described in Fig. 2 of the paper. Fig. S2 shows six particles in total, with two overlapping trajectories at a y-position of 100 μm. The overlapping trajectories for these cells can still be analyzed as they occur at different times.

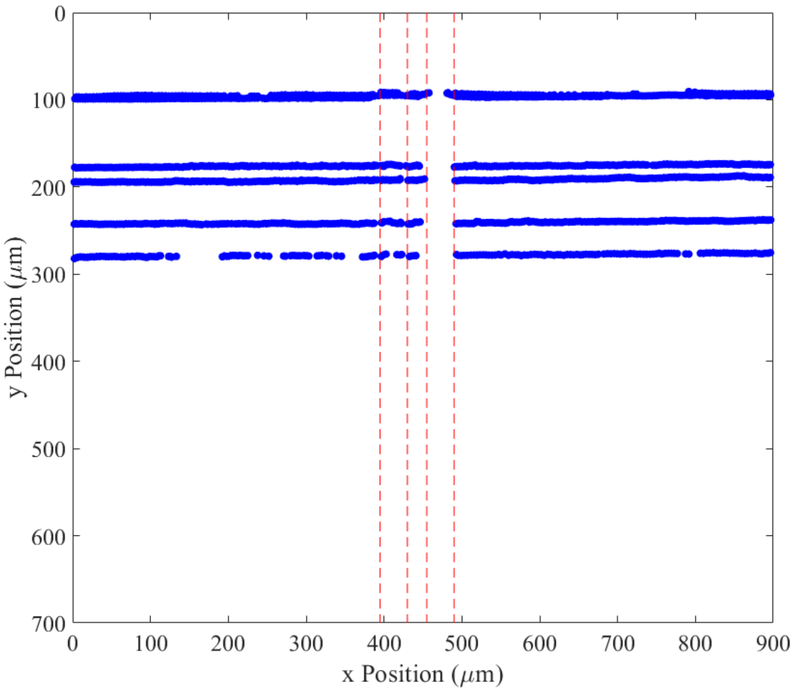

Fig. S2: Trajectories of detected cells. This figure illustrates the positions of cells within the field of view, with each cell tracked throughout the video to analyze motion in response to experimental conditions.

Experiment details for polystyrene spheres (PSS) analysis (Figure 4):

Figure 4 shows the differential velocity versus incoming velocity for a two-population mixture of 10 μm and 15.7 μm polystyrene microspheres (PSS) for a DEP voltage of 6 Vpp at f = 1 MHz. The data were obtained by analyzing the PSS particles from a 14-second imaging video. During measurements, the average flow rate was 1300 μm/s, with a PSS density of approximately 5.2×10^5 PSS/mL. The channel cross-section dimensions are 50 μm in height and 8000 μm in width, resulting in a volumetric flow rate of 0.52×10^{-3} mL/s. This corresponds to approximately 270 PSS/s traversing through the entire channel cross section. However, the imaging system field of view is 670x894 μm, resulting in an approximate 23 PSS/s throughput for imaging analysis. During the 14-second imaging video, 320 PSS were observed passing through the field of view, of which 175 were successfully tracked and analyzed to provide their input and output velocities (v_i and v_o) and differential velocity. The tracking and analysis efficiency was 55% in this case. Video S2 (PSS_Figure4_Video.mp4) in the supplementary material is a 5-second segment extracted from the original imaging video. This would be near the maximum particle density that could be tracked without dramatically decreasing the tracking efficiency.
